# Safety and Diagnostic Yield of Medical Pleuroscopy (MP) Performed under Balanced Analgosedation by a Pneumological Team Compared to Video-Assisted Thoracic Surgery (VATS): A Retrospective Controlled Real-Life Study (TORAPO)

**DOI:** 10.3390/diagnostics14060569

**Published:** 2024-03-07

**Authors:** Valentino Allocca, Luca Guidelli, Angela Galgano, Lucia Benedetti, Roberto Fabbroni, Andrea Bianco, Piero Paladini, Raffaele Scala

**Affiliations:** 1 Department of Translational Medical Sciences, “Luigi Vanvitelli” University of Campania, 80131 Naples, Italy; valentino.allocca@unicampania.it (V.A.); andrea.bianco@unicampania.it (A.B.); 2 Pulmonology and Respiratory Intensive Care Unit, S. Donato Hospital, Cardio-Thoraco-Neuro-Vascolar Department, Usl Toscana Sudest, 52100 Arezzo, Italy; guidelliluca6@gmail.com (L.G.); lucia.benedetti@uslsudest.toscana.it (L.B.); roberto.fabbroni@uslsudest.toscana.it (R.F.); 3 Thoracic Surgery Unit, Department of Medical, Surgical and Neuroscience Sciences, S. Maria Le Scotte Hospital, University of Siena, 53100 Siena, Italy; angela.galgano@student.unisi.it (A.G.); piero.paladini@unisi.it (P.P.)

**Keywords:** medical pleuroscopy, toracoscopy, diagnostic yield, analgosedation, propofol, pulmonologist

## Abstract

Introduction: Medical pleuroscopy (MP) is an invasive technique that provides access to the pleural space with a rigid or semi-rigid work instrument, allowing for visualization and the obtaining of bioptic pleural samples. Using pulmonologist-based analgosedation to perform pleuroscopy is still debated for safety reasons. The aim of this real-life study is to demonstrate the safety and diagnostic yield of MP performed under balanced analgosedation by a pulmonologist team with expertise in the management of critically ill patients in the respiratory intensive care unit (RICU) and interventional pulmonology unit as compared to video-assisted thoracic surgery (VATS) performed by a thoracic surgeon team under anesthesiologist-based analgosedation. Methods: In this multicentric retrospective controlled study, the inclusion criteria were patients older than 18 years old with pleural effusion of unknown diagnosis consecutively admitted in the years 2017–2022 to the pulmonology unit and RICU of San Donato Hospital in Arezzo (Italy, Tuscany) and to the thoracic surgery unit of Santa Maria Le Scotte in Siena (Italy, Tuscany) to undergo, respectively, MP under balanced propofol-based analgosedation on spontaneous breathing with local anesthesia provided by a pulmonologist team (Group A), and VATS provided by a surgeon team under propofol-based analgosedation managed by an anesthesiologist using invasive mechanical ventilation (IMV) via endotracheal intubation (ETI) (Group B). The primary endpoints were (1) a comparison between the two groups in terms of the diagnostic yield of pleural effusion, and (2) major and minor complications of pleuroscopic procedures. The secondary endpoints were (1) the length of the pleuroscopic procedure; (2) the duration of hospitalization; (3) propofol doses; and (4) the patient’s comfort after the procedure assessed using the Visual Analogue Scale (VAS). Results: We enrolled 91 patients in Group A and 116 patients in Group B. A conclusive diagnosis was obtained in 97.8% of Group A vs. 100% of Group B (*p* = 0.374). Malignant effusion was diagnosed in 59.3% of Group A and in 55.1% of Group B; *p* = 0.547. No intraoperative or postoperative mortality events or major complications were observed in Group A. The major complications observed in Group B were three major bleeding events (*p* = 0.079) and one exitus (*p* = 0.315) not related to the interventional procedure. No significant difference emerged between the two groups in terms of minor complications. The duration of the intervention was significantly lower in Group A (40.0 min ± 12.6 versus 51.5 ± 31.0; *p* = 0.001). Pain control and, therefore, patient comfort were better in Group A, with an average VAS of 0.34 ± 0.65 versus 2.58 ± 1.26, *p* < 0.001. The duration of hospitalization was lower in Group B (5.1 ± 2.6 vs. 15.5 ± 8.0, *p* < 0.001). The average overall dose of propofol administered was significantly lower in Group A (65.6 ± 35.8 mg versus 280 ± 20.0 mg; *p* < 0.001). Conclusions: This real-life study shows that the MP performed under propofol-based analgosedation by an independent pneumologist team is a safe and well-tolerated procedure with a diagnostic yield and complication rates similar to those obtained with VATS.

## 1. Introduction

Pleural diseases are common and increasing worldwide, affecting over 3000 people per 1 million each year [1]. Malignant pleural effusion, mesothelioma, and pleural infections represent an enormous burden and a clinical challenge for respiratory physicians, with approximately 361,270 hospitalizations occurring in the United States in 2016 [2]. For this reason, it is increasingly important to improve the current techniques for the diagnosis and management of pleural diseases. Medical pleuroscopy (MP), also known as medical thoracoscopy, is an invasive technique that provides access to the pleural space with a rigid or semi-rigid work instrument, which allows for the visualization and biopsy of pleural lesions [3]. In contrast to video-assisted thoracoscopy (VATS), which is performed by a surgeon under general anesthesia, often using single-lung ventilation and via multiple ports, MP is performed by a pulmonologist using analgosedation and spontaneous breathing via a single port [4]. Currently, there is not enough evidence to establish the diagnostic test effectiveness when comparing awake thoracoscopic pleural biopsy to video-assisted thoracoscopic pleural biopsy performed under general anesthesia [5]. In the latest guidelines of the British Thoracic Society, MP finds space in numerous applications for the diagnosis of pleural effusion, for example, obtaining pleural tissue sampling, which is often necessary to achieve a definitive diagnosis in patients with pleural effusion and/or pleural thickening [5]. The major indications for MP are (1) parietal pleural biopsies for the diagnosis of mesothelioma, lung cancer, and pleural infection, such as tuberculosis, which, in a significant majority of cases, can mimic a tumor; (2) debridement of simple adhesions in early-stage empyema; and (3) talc poudrage pleurodesis for malignant pleural effusion and in selected secondary pneumothorax not suitable for surgery [5,6]. The diagnosis of tuberculosis (TB) pleurisy becomes straightforward when Mycobacterium tuberculosis is identified in the sputum, pleural fluid, or biopsy samples. In regions with a high prevalence of TB, the combination of a lymphocyte-predominant exudate and elevated adenosine–deaminase (ADA) yields a positive predictive value of 98% [7]. In areas with low TB prevalence, the absence of increased ADA and lymphocyte predominance suggests a low likelihood of TB. In such cases, pleural biopsy is recommended to confirm the diagnosis. Additionally, when there is a high prevalence of drug-resistant TB, pleural biopsy for liquid culture and susceptibility testing should be considered [7]. According to the latest BTS guidelines, MP is the preferred method for obtaining pleural tissue sampling for culture and a sensitivity test for TB pleural effusion [8,9]. In addition, pleural biopsies allow for obtaining material on which to perform polymerase chain reaction (PCR) to detect viruses, like influenza, coxsackievirus, respiratory syncytial virus (RSV), cytomegalovirus, adenovirus, human herpesvirus-8, dengue, human t-lymphotropic virus type 1 (HTLV-1), varicella, herpes simplex virus (HSV), and Epstein–Barr virus (EBV), which can be responsible for pleural effusion [10]. MP represents a valid option as a first-line approach in the diagnosis of pleural diseases, offering potential advantages in comparison to VATS. These benefits include minor invasiveness; a reduction in costs to perform the procedure, as it can be performed in an endoscopic room with a shorter duration of hospitalization; the possibility of including frail patients who are at increased risk of intubation, mechanical ventilation, and general anesthesia. Overall, this approach results in a good diagnostic yield [3,6]. MP is a relatively safe procedure with a low mortality rate and low occurrence of major complications, such as hemorrhage, persistent air leaks, port tumor dissemination, and empyema, in comparison to VATS [6,11]. Minor complications, such as transient hypoxemia and subcutaneous emphysema, are less frequently observed in patients undergoing MP instead of VATS [4,6]. According to the majority of published data and clinical field experiences, moderate to severe analgosedation during pulmonologist interventional procedures is usually performed by anesthesiologists, especially if non-midazolam-based regimens are used.

The aim of this study is to demonstrate the safety and diagnostic yield of MP performed using analgosedation by a pneumologist team in collaboration with nurses with consolidated experience in the management of airways and respiratory-critical patients in the respiratory intensive care unit (RICU) and interventional pulmonology unit in a comparative analysis, with data obtained from a series of patients undergoing pleuroscopic examination using VATS performed by a thoracic surgeon team under anesthesiologist-based analgosedation [12].

## 2. Materials and Methods

In this multicentric retrospective controlled study, the inclusion criteria were patients older than 18 years with pleural effusion of unknown diagnosis consecutively admitted between 2017 and 2022 to Pulmonology and RICU of “San Donato” Hospital in Arezzo (Italy, Tuscany) and to the Thoracic Surgery Unit of “Santa Maria Le Scotte” in Siena (Italy, Tuscany). The patients underwent, respectively, MP under balanced propofol-based analgosedation on spontaneous breathing with local anesthesia provided by a pulmonologist team (Group A) and VATS provided by a surgical team under propofol-based analgosedation managed by an anesthesiologist in invasive mechanical ventilation (IMV) via endotracheal intubation (ETI) (Group B). The choice between the two procedures depended solely on the hospital where the patients were admitted. Specifically, patients admitted to “San Donato” Hospital underwent MP, while those admitted to “Santa Maria Le Scotte” Hospital underwent VATS.

The exclusion criteria were as follows: (1) terminally ill patients with a life expectancy of fewer than 6 months, (2) multiorgan failure [13], (3) active bleeding from thoracic and/or extrathoracic sources, (4) American Society of Anesthesiologists (ASA) score >3, (5) acute respiratory failure (ARF) requiring non-invasive and invasive mechanical respiratory support, (6) pregnancy, and (7) patient refusal. Informed consent was obtained from all recruited patients and the study protocol was approved by the local ethical committee (TORAPO 23776).

### 2.1. Medical Pleuroscopy (Group A)

Before the procedure, all patients underwent a chest X-ray followed by contrast-enhanced thoracic computerized tomography (CT) to better characterize and confirm the pleural disease. In some instances, positron emission tomography and computed tomography (PET-CT) scans were considered to aid in better identifying the target of biopsy on the pleural surface. Lung ultrasonography was performed in all patients using a convex probe (3.5–5 MHz) (Mindray Mobile Trolley UMT-160, Shenzhen, China) to define the extension and the features of the pleural effusion (i.e., septations and loculations); detect, at bedside, the point of chest access for instrument insertion; and study the motility of the diaphragm.

MP was performed in an endoscopic room under continuous assessment of heart rate (HR), respiratory rate (RR), blood pressure (BP), and three-lead ECG registered with a multiparametric monitor (Mindray Patient Monitor ePM 12, Shenzhen ,China). Full equipment for non-invasive respiratory support, including-high-flow nasal cannula (HFNC) and non-invasive ventilation (NIV), as well as for the management of airways and cardiopulmonary emergency (Guedel cannula, laryngeal mask, endotracheal tube, defibrillator) was available.

The pulmonologist team consisted of 2 respiratory physicians and 2 nurses with experience in airway management, in the management of critically ill respiratory patients, and in interventional procedures. In case of severe periprocedural complications, an anesthesiologist of the hospital was available on call.

Once the American Society of Anesthesiologists (ASA) score was established and informed consent was collected [14], the patients were placed in a lateral decubitus position with the administration of conventional oxygen by means of a Venturi mask titrated to achieve a target SpO_2_ of 94–98% and 90–92%, respectively, for hypoxemic and hypercapnic patients. An intravenous line, primed with 500 mL NaCl 0.9%, was set up to increase patients’ volume in case of propofol-induced arterial hypotension. Once the thoracoscope access point was identified using the ultrasound-guided mode, the local anesthetic of the intercostal cutaneous, subcutaneous, and pleural layers was performed with lidocaine 2% at a dosage of 200–300 mg.

The analgosedation procedure was set according to the protocol used in our previous study [15]. An initial intravenous dose of 1% propofol (0.5 mg/kg) was administered in a bolus followed by a continuous infusion maintenance dose of 0.5–1.0 mg/kg/h. A dose of intravenous meperidine (100 mg/2 mL) was administered at a dosage of 0.5 mg/kg. The administered drugs were then balanced to achieve a Richmond Agitation Sedation Scale (RASS) between −2 and −3 and VAS ≤ 2. The RASS is used to define the patient’s level of sensorium and to titrate the sedation according to predefined target values. Structured on a 10-point scale, it defines 4 levels of increasing agitation disturbances (from +1 to +4), a neutral level (0), and 5 levels of progressively depressed sensorium (from −1 to −5) [16]. In case of insufficient sedation, discomfort, or agitation, additional boluses of propofol (10–20 mg) were administered up to a maximum of 1 mg/kg.

**Figure 1:**
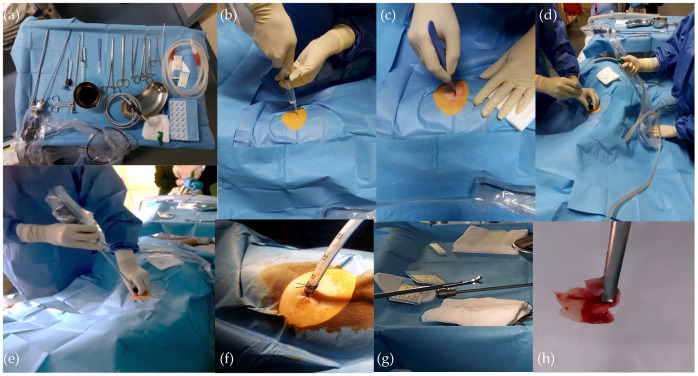
Medical pleuroscopy: an overview of the operation. From the top left corner: (**a**) instrumentation for MP; (**b**) local anesthetic injection; (**c**) Skin incision with a scalpel; (**d**) drainage of pleural effusion; (**e**) introduction of the optics; (**f**) chest tube drainage; (**g**) thoracoscope forceps; (**h**) pleural biopsy.

MP was performed with a rigid thoracoscope (Storz 4 mm, Karl Storz SE & Co. KG., Tuttlingen, Germany), and coagulation electrosurgery (Medtronic Covidien, Dublin, Ireland) was used to manage sources of bleeding. In general, 10–12 biopsies of the parietal pleura were performed. After pleuroscopy, a 20–24 Fr chest tube was placed at the thoracoscope access point (Figure 1).

In case of hypotension, the protocol involved the rapid infusion of crystalloids (500 mL of NaCl 0.9%); in case of a lack of response to volume filling, ephedrine hydrochloride (3 mg/mL) was administered intravenously at an initial dosage ranging between 3 and 6 mg, to be increased up to a maximum of 30 mg in total depending on the hemodynamic response. The goal was to maintain the mean arterial pressure (MAP) between 60 and 100 mmHg. In the event of bradycardia complicated by hypotension, the protocol allowed the reduction of the infusion rate of propofol to 0.5 mg/kg/h. Subsequently, atropine sulfate was administered intravenously at a dosage of 0.3–0.6 mg targeted at maintaining the HR between 60 and 90 bpm. In case of acute respiratory failure (ARF), defined as PaO_2_ < 60 mmHg with or without hypercapnia PaCO_2_ > 45 mmHg under conventional oxygen support, the protocol considered an escalating step-by-step strategy. This included a drop in the rate of propofol infusion to 0.5 mg/kg/h, followed by a jaw dislocation maneuver and the insertion of an oropharyngeal cannula (Guedel cannula) or a laryngeal mask in case of persistent airway collapse. Subsequently, the application of HFNC or NIV was considered until the escalation to endotracheal intubation (ETI) and IMV if mandatory respiratory support was needed.

During the MP in analgosedation, we continuously monitored patients’ SpO2, respiratory rate, heart rate, and blood pressure. Additionally, we frequently assessed the patients’ level of sedation to maintain an RASS between −2 and −3 (indicating light to moderate sedation). While there is strong clinical evidence supporting the use of capnography in general anesthesia and moderate to deep sedation [17], there is limited evidence in the context of light–moderate sedation in non-anesthesia settings in which the available evidence does not substantiate the impact of capnography on clinical outcomes when compared to standard monitoring [18]. Unfortunately, our equipment did not include a capnograph, so if there was any suspicion of ventilation problems, we performed arterial blood gas analysis.

The recovery time of the patient was evaluated according to the achievement of an Aldrete score ≥ 9 [15]. The Aldrete score is an extensively validated scoring scale used to establish safe post-anesthetic discharge in a hospital ward or at home for patients undergoing short-term surgical procedures [19]. Once the Alderete score was ≥9 after the procedure, the patients were discharged from the endoscopic room and admitted to the monitored beds of Pulmonology and the RICU [19]. Two hours later, a chest X-ray was performed as well as bloodwork (i.e., chemistry, blood cell count) [13,14].

### 2.2. VATS (Group B)

Patients belonging to Group B underwent the same preliminary clinical, radiological, and ultrasound-based assessment as described for Group A.

In Group B, VATS was performed with a uniportal approach by a surgeon. The procedure took place after the patient was endotracheally intubated and mechanically ventilated under general anesthesia, managed by the anesthesiologist team in the operating room. The anesthetic protocol included the use of propofol 1% at 1.5–2 mg/kg intravenously administered in a bolus followed by a continuous infusion at 3–4 mg/kg/h. Fentanyl (0.1 mg/2 mL) was administered intravenously at a dosage of 1 mcg/kg as a bolus followed by an infusion of Remifentanil at 0.1 mcg/kg/h. VATS was performed with a rigid thoracoscope (Storz 10 mm) and coagulation electrosurgery (Covidien) was used to manage sources of bleeding. An endopleural drainage (24–28 Fr) was placed at the end of the procedure. Complications were managed by the anesthesiologist team according to protocols similar to those reported for Group A.

Once the Alderete score was ≥9 after the procedure, the patients were discharged from the endoscopic room and admitted to the monitored beds of the Thoracic Surgery Unit.

### 2.3. Endpoints of the Study

The primary endpoints of the study were the comparative rates between the two groups in terms of (1) the diagnostic yield of pleural effusion and (2) the major and minor complications of pleuroscopic procedures.

The major complications considered in the study were (1) ARF requiring ventilator support with HFN (high-flow nasal cannula), NIV, and/or ETI with IMV; (2) acute coronary syndrome and/or cardiogenic pulmonary edema; (3) massive bleeding; and (4) periprocedural death. Bleeding was defined as either a loss of more than one blood volume in 24 h or 50% of the total blood volume of the patient in 3 h, or blood loss > 150 mL/min [20,21].

The minor complications considered in the study were new-onset cardiac arrhythmias requiring treatment, subcutaneous emphysema, hypoxemia defined as SpO_2_ < 90% for more than 1 min in conventional oxygen therapy at FiO2 of 0,50, arterial hypotension defined as systolic blood pressure (SBP) < 90 mmHg or diastolic blood pressure (DBP) < 50 mmHg in three consecutive measurements requiring volume filling and/or the use of vasoactive amines, bradycardia defined as heart rate (HR) < 50 bpm for more than two minutes and arterial hypertensive crisis defined as SBP > 170 mmHg or DBP > 100 mmHg in three consecutive measurements and requiring hypotensive therapy.

The secondary endpoints of the study were the comparative assessment of the two groups in terms of (1) the length of pleuroscopic procedure, (2) the duration of hospitalization, (3) propofol doses, and (4) the patient’s comfort after the procedure assessed using the Visual Analogue Scale (VAS).

## 3. Statistical Analysis

The statistical analysis of the data was carried out using Microsoft Excel v. 16.78.3 and the statistical software MiniTab v. 21.4.1. The data are expressed as the mean with standard deviation (SD), or as the median with interquartile range (IQR) for continuous variables and numbers with percentages for categorical variables. The Student’s *t*-test was used to compare continuous variables between the group undergoing MP and the group undergoing VATS. The chi-square test was used to compare the categorical variables between the two groups. A value of *p* < 0.05 was considered statistically significant.

## 4. Results

During the study time, 91 patients were enrolled in Group A and 116 patients in Group B. The baseline clinical features of the two groups are presented in Table 1. The two groups were similar for all reported parameters except for the Charlson Comorbidity Index, which was significantly greater in Group A compared to Group B (4.27 ± 2.04 versus 1.64 ± 1.52, *p* < 0.001).

### 4.1. Primary Endpoints

The diagnostic yield rate did not differ between the two groups (Group A: 97.8%, Group B: 100%; *p* = 0.374). Table 2 reports the etiologic diagnosis obtained in the groups; in both groups, malignancies accounted for the greater amount of underlying pleural effusion (Group A: 59.3%, Group B: 55.1%; *p* = 0.547). Among non-malignant etiologies, the most common diagnosis was non-specific pleuritis. No major complications, either intra- or postoperative, were observed in Group A. The major complications observed in Group B were three cases of major bleeding (*p* = 0.079) and one exitus (*p* = 0.315), which occurred due to intestinal infarction after some days and was, therefore, not connected to the interventional procedure. In the comparison between the two groups, the minor complications were superimposed without any statistically significant difference. Specifically, we observed two cases of hypotension in Group A and two in Group B (*p* = 0.809); two cases of bradycardia during the procedure in Group A and none in Group B (*p* = 0.153); one hypertensive crisis in Group A and none in Group B (*p* = 0.315); and no cases of atrial fibrillation in Group A and one case in Group B (*p* = 0.315). Post-procedure subcutaneous emphysema was observed in four patients in Group A and eight patients in Group B without significant differences (*p* = 0.433). No case of hypoxemia requiring respiratory support was observed in either group (Table 3). In five cases in Group A, no biopsy could be performed due to the presence of tenacious adhesions, despite initial debridement with digitoclasia; these patients were then sent to VATS. In two cases belonging to Group A, the samples obtained were not sufficient for diagnosis (indetermined etiologies in Table 2).

### 4.2. Secondary Endpoints

**Figure 2:**
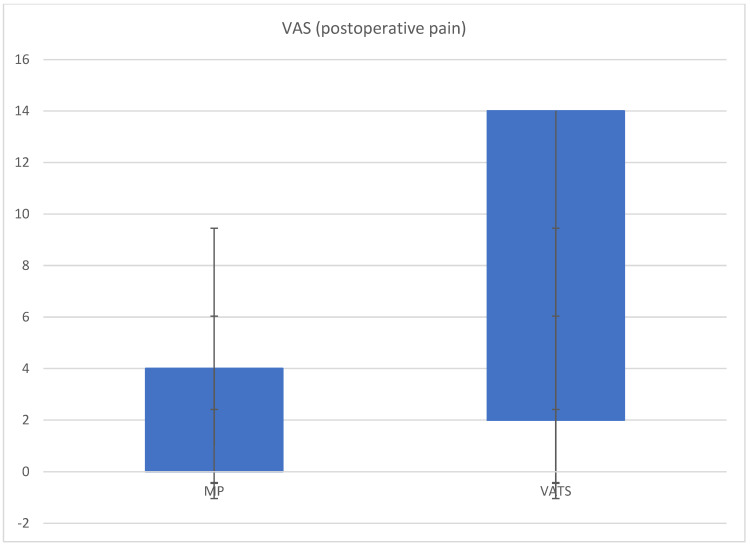
Postoperative pain in MP versus VATS using Visual Analogue Scale (VAS) (IQR 0–3, median 0, Group A) (IQR 0–6, median 3, Group B).

**Figure 3:**
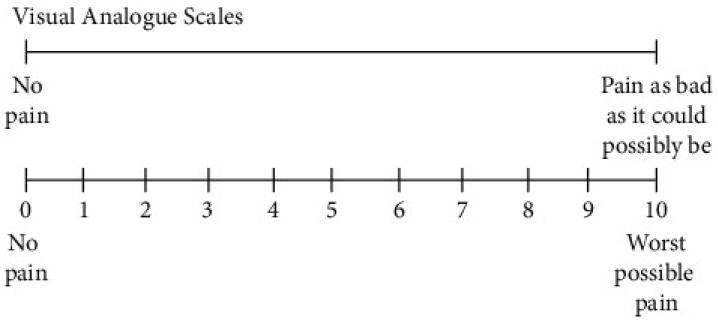
Visual Analogue Scale (VAS) used for evaluating postoperative pain.

In the comparison between the two groups, the duration of intervention was significantly lower in Group A (40.0 min ± 12.6 versus 51.5 ± 31.0; *p* = 0.001). Pain control and, therefore, patient comfort were better in Group A with an average VAS of 0.34 ± 0.65 versus 2.58 ± 1.26, *p* < 0.001 (Figure 2 and Figure 3). The duration of hospitalization was lower in Group B than in Group A (5.1 ± 2.6 vs. 15.5 ± 8.0, *p* < 0.001). The average overall dose of propofol administered was significantly lower in Group A than in Group B (65.6 ± 35.8 mg versus 280 ± 20.0 mg; *p* < 0.001). The average dose of meperidine administered was 46.2 ± 27.0 mg, while the average dose of fentanyl administered was 70 ± 10 mcg.

## 5. Discussion

The aim of this controlled study was to demonstrate the safety and diagnostic yield of MP performed in analgosedation by a pulmonologist team with established experience in airway management and respiratory critically ill patients in an RICU and interventional procedures, in a comparative analysis with data obtained from a series of patients who underwent pleuroscopic examination in VATS by a surgeon team under anesthesiologist-based management. The diagnostic yield was over 97% and it was not significantly different compared to that obtained in the VATS group (100%). In our study, in both groups, malignancies accounted for the greater amount of underlying pleural effusion (Group A: 59.3%, Group B: 55.1%; *p* = 0.547). Among the non-malignant etiologies, the most common diagnosis was that of non-specific pleuritis, described as fibrinous or inflammatory pleuritis without a specific attributable benign or malignant cause, but in subsequent follow-ups, proved to be benign in 85% of cases [22]. Another important finding to highlight is the diagnosis of TB, identified in two cases in Group A and four in Group B. After obtaining the diagnosis through PCR analysis on the biopsy sample, subsequent culture and antibiotic sensitivity testing was performed.

No deaths and no major complications, either intraoperative or postoperative, were observed in the MP group. These results are in line with literature [23,24,25,26]. In the retrospective observational study of Valsecchi et al. on 2752 analyzed MPs, the diagnostic yield was about 80%, and in more than half of the cases, the diagnosis was consistent with malignancies. The most frequent tumor was mesothelioma (about 21%), followed by metastases of breast cancer (14%). Tubercular pleurisy was the most frequent diagnosis in non-malignant pleural effusion (about 6% of cases) [23]. In Valsecchi’s study, it was also estimated that MP obtained a good diagnostic yield in case of monolateral pleural effusion, but dropped to about 50% in the case of bilaterality of the effusion. These findings underline that the good selection and study of the patient to be subjected to the procedure are essential to achieve an accurate diagnosis [23]. In our study, we identified two cases of pleural effusion secondary to heart failure. The diagnosis of heart failure was subsequently made based on clinical and laboratory criteria, such as NT-proBNP. These were two cases of unilateral pleural effusion with no evidence of other pathology found in the pleural biopsies performed. Improvement in the effusion with the optimization of medical therapy confirmed the diagnostic suspicion. In another study of 19 patients who underwent MP, the diagnosis was obtained in 69% of cases (13/19 patients) and malignant etiology was observed in 100% of cases [24]. A diagnostic yield of 97.8%, very similar to that reported in our experience, was obtained in the study of Dhooria et al. conducted on 145 patients with pleural effusion undergoing MP with a rigid thoracoscope. In the same study, the use of the rigid thoracoscope was found to be superior to the use of the semi-rigid instrument [25].

Also, in a recent meta-analysis on the complications of thoracentesis and MP performed for malignant pleural effusion, the complication rate was only 0.040 (95% CI 0.029–0.052) [26].

In the British Thoracic Society (BTS) guidelines for pleural diseases, MP with a rigid thoracoscope is considered a valid procedure as well as one performed in VATS with no difference in diagnostic yield, sensitivity, or specificity [8]. Also, in the Clinical Statement of the BTS on pleural procedures, MP performed with local anesthetic is considered a safe procedure with a death rate of 0.3% [8,27]. Major complications such as massive bleeding, pleural infections, and pneumothorax occur in only 1.8% of cases, while minor complications such as subcutaneous emphysema, atrial fibrillation, mild bleeding, hypotension, and fever occur in 7.8% [8,27]. Other studies confirm the safety of MP by establishing that complications are rare, with a mortality between 0.09% and 0.24% [28,29,30]. In another study with the objective of evaluating MP in patients at high risk of complications, mortality was 0.28% with minor complications such as postoperative pain in 12.3% of cases and subcutaneous emphysema in 10.3%. No complications were observed in 58.9% of cases, thus establishing the safety of the procedure in high-risk patients [31]. Mild to moderate pain and minor bleeding were described in another study of 14 patients with an overall diagnostic yield of 100% [32].

In our study, in the MP group, we observed a lower, statistically insignificant rate of major bleeding compared to the VATS group and there was no statistically significant difference in terms of other complications between the two groups. The trend towards a reduction in bleeding in the MP group may be due to the use of a smaller instrument (Storz 4 mm vs. 10 mm) and, therefore, of smaller biopsy samples and smaller thoracostomy access.

The inpatient time was statically longer in patients from Group A than Group B, and this is probably due to the worse clinical conditions of patients enrolled in Group A with a Charlson Comorbidity Index of 4.27 ± 2.04 versus 1.64 ± 1.52 in Group B (*p* < 0.001). In addition, the patients in Group B were part of a “fast-track” path for pleural effusion with hospitalization aimed at pleuroscopic intervention.

The pain was statistically better controlled in the group subjected to MP than in Group B. The reasons for the reduction in pain are likely due to the minor invasiveness of MP, the smaller size of the instrument, and, consequently, the smaller thoracostomy access [33]. The ability to perform the procedure without orotracheal intubation drastically improves patient comfort and recovery time [33]. Performing a pleuroscopy under analgosedation and spontaneous breathing also allows the enrolment of patients with multiple comorbidities who would usually be excluded from more invasive procedures.

Another advantage of our study was that MP was performed under analgosedation by a pulmonologist team without the support of the anesthesiologist. Currently, in Italy and in all European countries, the administration of propofol is authorized not only for anesthesiologists, but for all physicians with extensive experience in managing patients in intensive care units [34]. The use of a propofol-based regimen for analgesia and sedation is contemplated in the guidelines “Practice Guidelines for Sedation and Analgesia by non-Anesthesiologists”, suggesting that when used for moderate sedation, the use of propofol or ketamine offers satisfactory results [35,36]. The same guidelines also suggest that when propofol is used for moderate sedation, the user should be able to intervene with rescue therapy at all levels of sedation, including general anesthesia [35,36]. Pulmonologists working in an RICU have both the experience and the knowledge in the management of possible cardio-respiratory complications induced by propofol [15]. Of course, the role of nursing staff in monitoring the patient during the procedure and in supporting pneumologists throughout the procedure must be emphasized. The pulmonologist team of the “San Donato” Hospital of Arezzo related to Pneumology and the RICU has gained significant experience in analgosedation with protocols based on propofol and meperidine after completing a specific course aimed at developing both theoretical and practical knowledge on the use of drugs for sedation and the management of potential cardio-respiratory complications. Each pulmonologist (both physicians and nurses) of the team is capable in the management of the airways and in the active support of critical patients, even with invasive mechanical ventilation; moreover, the entire team is trained in the positioning of pleural drainage of all kinds for the management of pleural pathologies [15]. All of these competences are, therefore, indispensable in preventing and promptly intervening in the event of a possible complication. It is important to note that the anesthesiologist is always available in case of any complications in an integrated path between the RICU and ICU (intensive care unit).

Analgosedation managed by a pulmonologist team is also well established in the UK. As reported in the guidelines “BTS clinical statement on pleural procedures”, sedation is the responsibility of the thoracoscopist and is usually performed with the intravenous infusion of benzodiazepines (e.g., midazolam) and opioids (e.g., fentanyl). The anesthesiologist is generally involved in more complex sedations [8,27]. In real life, there is often fear regarding the use of propofol as an anesthetic for sedation and benzodiazepines are usually preferred by non-anesthesiologist teams [37]. This reluctance must be overcome, because there is already abundant literature data attesting to the safety of propofol use and it is considered, in many cases, to be superior in sedation compared to midazolam [38]. In the study conducted by Roekaerts et al. in which sedation with propofol and midazolam were compared after coronary surgery, during sedation in all patients, the hemodynamic parameters were stable except for a slight drop in systemic blood pressure for the propofol group and an increase in heart rate for the midazolam group [38], and the hemodynamic effects of both midazolam and propofol usually have no clinical significance [39]. In the work of Tschopp et al., balanced sedation with propofol was used in MP. The same authors showed that MP performed in analgosedation with propofol by non-anesthesiologist teams is a procedure that can be safely conducted without major cardio-pulmonary complications [40]. Complications such as hypoxemia and hypotension can be easily and quickly corrected in most cases [40]. In another randomized clinical trial, it appears that sedation with propofol performed by endoscopists gives the patient better satisfaction than deep sedation performed by anesthesiologists [41]. In addition, patients receiving sedation from non-anesthesiologists required significantly lower doses of propofol than those receiving sedation from anesthesiologists (94 mg versus 260 mg), with fewer side effects with statistically significant values [42,43]. Also, in our study, the doses of propofol administered were lower than those used in Group B, where sedation was the prerogative of the anesthesiologists (65 mg versus 280 mg), in the absence of major cardiorespiratory complications and with only two cases of hypotension in the 91 procedures carried out. The reduction in the dose of the sedative used obviously involves faster recovery of the patient and a lower risk of hemodynamic and respiratory side effects [44]. The reduction in the dosage of sedatives also did not affect pain control or patient comfort. In our study, in Group A, which involved a significantly lower dosage of anesthetics than Group B, the pain was better controlled with an average VAS of 0.34 ± 0.65 versus 2.58 ± 1.26.

This confirms the safety in the use of propofol as a sedative agent of choice compared to midazolam. In another non-inferiority randomized clinical trial that compared sedation with propofol in digestive endoscopic procedures performed by non-anesthetists and anesthesiologists in low-risk patient groups (ASA 1-2), the rate of complications (39%) was the same between the two groups without differences in the dose of propofol administered and without differences in amnesia and recovery time [41]. In a recent study carried out by Maffucci et al., the authors showed that sedation with propofol administered by a pneumologist team during bronchoscopic procedures was a safe practice without serious side effects. Complications occurred in only 25% of cases and were all successfully treated in the endoscopic room. Moderate sedation was achieved in 92% of subjects treated with adequate comfort and tolerance and with a recovery time of about 5–10 min on average [15]. Sedation based on propofol is now an integral part of the guidelines of gastrointestinal endoscopy with a frequency of adverse events similar to that of traditional sedation, but with a shorter recovery and duration of hospitalization [43,45]. Unlike endoscopic digestive procedures, in which the use of propofol for sedation performed by non-anesthesiologists is now consolidated [45], there is a lack of data and randomized clinical trials in the literature that clearly demonstrate the safety of sedation performed by pulmonologists in the diagnosis of pleural diseases. It is necessary, however, to highlight that the support of the anesthesiologist is indispensable in procedures of the highest risk (ASA 4) and that good collaboration between pulmonologists and anesthesiologists should be achieved.

Our study has some limitations that need to be considered. First, there was no control group in which sedation was performed by the same pulmonologist team, but with a regime based on midazolam. Second, the retrospective design of the study intrinsically has some biases like the risk of losing some relevant points such as the costs of the procedure and the real patient comfort. Finally, the study was conducted by a team with extensive experience in analgosedation with propofol and with excellent skills in airway management including invasive mechanical ventilation. The same results, therefore, may not be comparable to those of other centers with less expertise.

## 6. Conclusions

In summary, this study shows that MP performed under analgosedation by a pulmonologist team is a safe and well-tolerated procedure with a diagnostic yield similar to that obtained in VATS. The use of a sedation regimen with propofol and meperidine turns out to be safe with few side effects.

Unfortunately, there are few pulmonology units that routinely perform MP (estimated at approximately 30%) [46], and there are still too few pneumologists who deal first-hand with sedation. The purpose of this study was also to provide objective safety data so that this procedure becomes widely used in all pulmonology units. Too often, the management of pleural pathology is left to the thoracic surgeon, but pulmonologists must regain centrality in the management of these pathologies that increasingly present themselves in practice.

## Figures and Tables

**Table 1:** Characteristics of patients undergoing pleuroscopy; ASA: American Society of Anesthesiologists; COPD: chronic obstructive pulmonary disease; CAD: coronary artery disease; OSA: obstructive sleep apnea; CRF: chronic respiratory failure; DM: diabetes mellitus; SAH: systemic arterial hypertension; CKD: chronic kidney disease; ILD: interstitial lung disease. Data are presented as mean and standard deviation or absolute number (percentage).

	Group A	Group B	*p*
Patients, *n*	91	116	
Age, years (SD)	72 (11)	69 (11)	0.102
BMI (SD)	30 (8.72)	28 (7.45)	0.083
Male	62	79	0.997
Female	29	31	0.420
Charlson Comorbidity Index (SD)	4.27 (2.04)	1.64 (1.52)	<0.001
SpO2 % basal	92.44%	93.07%	0.138
ASA 3, *n* (%)	48 (50%)	46 (39.66%)	0.059
ASA 2, *n* (%)	48 (50%)	70 (60.34%)	0.273
COPD, *n* (%)	21 (23.07%)	7 (6.04%)	0.001
CAD, *n* (%)	20 (21.97%)	16 (13.79%)	0.129
OSA, *n* (%)	4 (4.39%)	0	0.041
CRF *n* (%)	2 (2.19%)	5 (4.31%)	0.385
DM, *n* (%)	12 (13.18%)	29 (25%)	0.028
Arrhythmias, *n* (%)	20 (21.97%)	16 (13.79%)	0.129
SAH, *n* (%)	49 (53.84%)	0	<0.001
Heart failure, *n* (%)	6 (6.59%)	13 (11.2%)	0.239
CKD, *n* (%)	9 (9.89%)	5 (4.31%)	0.127
Neoplasm, *n* (%)	28 (30.76%)	41(35.34%)	0.486
Stroke, *n* (%)	6 (6.59%)	8 (6.89%)	0.931
ILD, *n* (%)	3 (3.29%)	1 (0.86%)	0.237

**Table 2:** Etiologic diagnosis underlying pleural effusion obtained in the groups. NSCLC: non-small-cell lung cancer; TB: tuberculosis; SCLC: small-cell lung cancer. Data are presented as absolute values (percentages).

Diagnosis	Group A *n* (%)	Group B *n* (%)	*p*
**Malignant etiologies**	54 (59.3%)	64 (55.1%)	0.547
NSCLC	20 (22.0%)	17 (14.7%)	0.179
Metastases	14 (15.4%)	15 (12.9%)	0.671
Mesothelioma	17 (18.7%)	29 (25,0%)	0.270
SCLC	0	1 (0.9%)	0.315
Lymphoma	3 (3.3%)	1 (0.9%)	0.237
Hemangioendothelioma	0	1 (0.9%)	0.315
**Non-malignant etiologies**	30 (32.9%)	51 (43.9%)	0.103
Non-specific pleuritis	16 (17.6%)	39 (33.6%)	0.007
Empyema and parapneumonic effusion	10 (10.9%%)	4 (3.4%)	0.041
TB	2 (2.2%)	4 (3.4%)	0.585
Heart failure	2 (2.2%)	0	0.153
Asbestosis	0	1 (0.9%)	0.315
Sarcoidosis	0	2 (1.72%)	0.154
**Indetermined etiologies**	2 (2.1%)	0 (0%)	0.153

**Table 3:** Complications of medical pleuroscopy in analgosedation with pneumological management versus surgical pleuroscopy complications with anesthesiologic support. Spo2: peripheral oxygen saturation. Data are presented as absolute values (percentages). N/A: not applicable.

	Group A *n* (%)	Group B *n* (%)	*p*
**Major complications**	0 (0%)	4 (3.44%)	0.042
**Need for ventilatory support**	0 (0%)	N/A	1.000
**Major bleeding**	0 (0%)	3 (2.58%)	0.079
**Exitus**	0 (0%)	1 (0.86%)	0.315
**Minor complications**	9 (9.9%)	11(9.5%)	0.922
**Hypotension**	2 (2.19%)	2 (1.72%)	0.809
**Bradycardia**	2 (2.19%)	0 (0%)	0.153
**Subcutaneous emphysema**	4 (4.39%)	8 (6.89%)	0.433
**Hypoxemia (SpO2 < 90%)**	0 (0%)	0 (0%)	1.000
**Hypertensive crisis**	1 (1.09%)	0 (0%)	0.315
**Cardiac arrhythmias**	0 (0%)	1 (0.86%)	0.315

## Data Availability

The data presented in this study are available on request from the corresponding author due to privacy reasons.
